# Supplementary material for: Dynamics and mechanism of dimer dissociation of photoreceptor UVR8
Source: Nat Commun. 2022 Jan 10;13:93. doi: 10.1038/s41467-021-27756-w (PMC8748919; doi:10.1038/s41467-021-27756-w)
Supplement: Supplementary file 1 — Supplementary Information [file 41467_2021_27756_MOESM1_ESM.pdf]

# Supplementary Materials for

## Dynamics and mechanism of dimer dissociation of photoreceptor UVR8

Xiankun Li,[1,2] Zheyun Liu,[1] Haisheng Ren,[3,4] Mainak Kundu,[1] Frank W. Zhong,[5] Lijuan Wang,[1] Jiali Gao,[3,6]* Dongping Zhong[1,2]*

Correspondence to: jiali@jialigao.org and zhong.28@osu.edu

**Affiliations:**

[1]Department of Physics, Department of Chemistry and Biochemistry, Programs of Biophysics, Chemical Physics and Biochemistry, The Ohio State University, Columbus, Ohio 43210, United States. [2]Center for Ultrafast Science and Technology, School of Physics and Astronomy, School of Chemistry and Chemical Engineering, Shanghai Jiao Tong University, Shanghai 200240, China. [3]Department of Chemistry and Supercomputing Institute, University of Minnesota, Minneapolis, Minnesota 55455, United States. [4]College of Chemical Engineering, Sichuan University, Chengdu 610065, China. [5]Cell and Molecular Biology Program, University of Chicago, Chicago, Illinois 60637, United States. [6]School of Chemical Biology and Biotechnology, Peking University Shenzhen Graduate School, Shenzhen 518055, China.

**This PDF file includes:**

Supplementary Methods
Supplementary Text
Supplementary Tables 1-3
Supplementary Figures 1-14
Supplementary Reference List

**Supplementary Methods**

**UVR8 DNA sequences.**

UVR8 full length
ATGGCGGAGGATATGGCTGCCGACGAAGTTACGGCTCCTCCTCGTAAGGTTCTTATCATCTC
CGCTGGTGCTAGCCACTCCGTCGCTCTTCTCTCTGGTGACATTGTTTGTTCTTGGGGTCGAGG
AGAGGATGGACAGTTAGGTCATGGCGATGCAGAGGATCGACCTTCTCCGACTCAGCTTAGC
GCTTTAGATGGCCACCAAATTGTTTCCGTTACCTGTGGTGCTGATCACACTGTTGCTTATTCA
CAATCAGGCATGGAAGTCTACAGTTGGGGATGGGGTGATTTTGGGAGATTAGGCCATGGTA
ACTCAAGCGACTTGTTTACTCCGCTACCAATCAAAGCATTGCACGGTATTCGGATCAAGCAG
ATTGCTTGTGGGGATAGTCATTGTTTGGCTGTCACTATGGAAGGAGAGGTCCAGAGTTGGGG
CCGCAACCAGAATGGTCAACTTGGTCTGGGGGACACCGAAGATTCTCTAGTGCCTCAGAAG
ATTCAAGCCTTTGAGGGAATACGAATCAAAATGGTTGCTGCTGGTGCAGAACACACTGCTGC
AGTTACAGAAGATGGTGACCTCTATGGATGGGGCTGGGGAAGATACGGAAATTTGGGATTA
GGTGACCGGACTGACCGCTTAGTTCCTGAAAGAGTTACCTCTACTGGTGGTGAGAAAATGTC
AATGGTTGCTTGTGGATGGCGGCACACAATATCAGTTTCCTACTCTGGAGCATTGTATACTTA
TGGATGGAGCAAATATGGACAGCTAGGACATGGAGACTTGGAGGATCACCTTATTCCTCAC
AAACTGGAAGCACTGAGCAACAGTTTTATCTCCCAGATTTCGGGAGGTTGGAGACATACAAT
GGCATTGACTTCAGATGGAAAACTATATGGATGGGGTTGGAATAAGTTTGGACAAGTAGGA
GTCGGCAATAATTTAGATCAGTGTTCTCCTGTGCAAGTGCGATTTCCCGATGATCAGAAAGT
AGTTCAAGTCTCATGTGGATGGAGACATACCTTGGCTGTCACTGAAAGAAATAACGTGTTTG
CTTGGGGTAGAGGTACAAATGGACAGCTCGGCATTGGAGAGTCGGTTGACAGGAACTTTCC
CAAGATTATAGAGGCACTCAGCGTCGATGGAGCAAGTGGACAACATATAGAATCTTCTAAT
ATCGATCCATCTTCAGGGAAAAGCTGGGTGTCGCCTGCAGAGAGATATGCAGTTGTTCCTGA
TGAAACGGGCCTAACGGATGGTTCAAGCAAAGGTAATGGAGGTGATATCAGTGTTCCACAA
ACTGATGTCAAGCGTGTACGAATTTGA

UVR8 deltaC (codon optimized)
CATATGGCGGAGGATATGGCTGCCGACGAAGTTACGGCTCCTCCTCGTAAGGTTCTTATCATCT
CCGCTGGTGCTAGCCACTCCGTCGCTCTTCTCTCTGGTGACATTGTTTGTTCTTGGGGTCGAG
GAGAGGATGGACAGTTAGGTCATGGCGATGCAGAGGATCGACCTTCTCCGACTCAGCTTAGC
GCTTTAGATGGCCACCAAATTGTTTCCGTTACCTGTGGTGCTGATCACACTGTTGCTTATTCAC
AATCAGGCATGGAAGTCTACAGTTGGGGATGGGGTGATTTTGGGAGATTAGGCCATGGTAACT
CAAGCGACTTGTTTACTCCGCTACCAATCAAAGCATTGCACGGTATTCGGATCAAGCAGATTG
CTTGTGGGGATAGTCATTGTTTGGCTGTCACTATGGAAGGAGAGGTCCAGAGTTGGGGCCGC
AACCAGAATGGTCAACTTGGTCTGGGGGACACCGAAGATTCTCTAGTGCCTCAGAAGATTCA
AGCCTTTGAGGGAATACGAATCAAAATGGTTGCTGCTGGTGCAGAACACACTGCTGCAGTTA
CAGAAGATGGTGACCTCTATGGATGGGGCTGGGGAAGATACGGAAATTTGGGATTAGGTGAC
CGGACTGACCGCTTAGTTCCTGAAAGAGTTACCTCTACTGGTGGTGAGAAAATGTCAATGGTT
GCTTGTGGATGGCGGCACACAATATCAGTTTCCTACTCTGGAGCATTGTATACTTATGGATGGA
GCAAATATGGACAGCTAGGACATGGAGACTTGGAGGATCACCTTATTCCTCACAAACTGGAAG
CACTGAGCAACAGTTTTATCTCCCAGATTTCGGGAGGTTGGAGACATACAATGGCATTGACTT
CAGATGGAAAACTATATGGATGGGGTTGGAATAAGTTTGGACAAGTAGGAGTCGGCAATAATT
TAGATCAGTGTTCTCCTGTGCAAGTGCGATTTCCCGATGATCAGAAAGTAGTTCAAGTCTCAT
GTGGATGGAGACATACCTTGGCTGTCACTGAAAGAAATAACGTGTTTGCTTGGGGTAGAGGTA
CAAATGGACAGCTCGGCATTGGAGAGTCGGTTGACAGGAACTTTCCCAAGATTATAGAGGCA
CTCAGCGTCGATTAAGGATCC

**Primer sequences (UVR8 full length).**
The three-letter codon corresponding to the mutated amino acid residue(s) is highlighted in red. For each primer pair, the sequence is shown from 5' to 3'.

W39F
Top 5'-GGT GAC ATT GTT TGT TCT TTC GGT CGA GGA GAG GAT GGA C
Bottom 5'-G TCC ATC CTC TCC TCG ACC GAA AGA ACA AAC AAT GTC ACC

W92F
Top 5'-GGC ATG GAA GTC TAC AGT TTC GGA TGG GGT GAT TTT GGG
Bottom 5'-CCC AAA ATC ACC CCA TCC GAA ACT GTA GAC TTC CAT GCC

W94F
Top 5'-G GAA GTC TAC AGT TGG GGA TTC GGT GAT TTT GGG AGA TTA GGC
Bottom 5'-GCC TAA TCT CCC AAA ATC ACC GAA TCC CCA ACT GTA GAC TTC C

W144F
Top 5'-G GAA GGA GAG GTC CAG AGT TTC GGC CGC AAC CAG AAT GGT C
Bottom 5'-G ACC ATT CTG GTT GCG GCC GAA ACT CTG GAC CTC TCC TTC C

W196F
Top 5'-GAT GGT GAC CTC TAT GGA TTC GGC TGG GGA AGA TAC GG
Bottom 5'-CC GTA TCT TCC CCA GCC GAA TCC ATA GAG GTC ACC ATC

W198F
Top 5'-GGT GAC CTC TAT GGA TGG GGC TTC GGA AGA TAC GGA AAT TTG GG
Bottom 5'-CC CAA ATT TCC GTA TCT TCC GAA GCC CCA TCC ATA GAG GTC ACC

W233F
Top 5'-G TCA ATG GTT GCT TGT GGA TTC CGG CAC ACA ATA TCA GTT TC
Bottom 5'-GA AAC TGA TAT TGT GTG CCG GAA TCC ACA AGC AAC CAT TGA C

W233Y
Top 5'-CA ATG GTT GCT TGT GGA TAC CGG CAC ACA ATA TCA GTT TCC
Bottom 5'-GGA AAC TGA TAT TGT GTG CCG GTA TCC ACA AGC AAC CAT TG

W250F
Top 5'-GGA GCA TTG TAT ACT TAT GGA TTC AGC AAA TAT GGA CAG CTA GG
Bottom 5'-CC TAG CTG TCC ATA TTT GCT GAA TCC ATA AGT ATA CAA TGC TCC

W285F
Top 5'-CC CAG ATT TCG GGA GGT TTC AGA CAT ACA ATG GCA TTG AC
Bottom 5'-GT CAA TGC CAT TGT ATG TCT GAA ACC TCC CGA AAT CTG GG

W285Y
Top 5'-CC CAG ATT TCG GGA GGT TAC AGA CAT ACA ATG GCA TTG AC

Bottom 5’-GT CAA TGC CAT TGT ATG TCT GTA ACC TCC CGA AAT CTG GG

W300F
Top 5’-CA GAT GGA AAA CTA TAT GGA TTC GGT TGG AAT AAG TTT GGA CAA GTA GG
Bottom 5’-CC TAC TTG TCC AAA CTT ATT CCA ACC GAA TCC ATA TAG TTT TCC ATC TG

W300H
Top 5’-CA GAT GGA AAA CTA TAT GGA CAC GGT TGG AAT AAG TTT GGA CAA GTA GG
Bottom 5’-CC TAC TTG TCC AAA CTT ATT CCA ACC GTG TCC ATA TAG TTT TCC ATC TG

W300L
Top 5’-CA GAT GGA AAA CTA TAT GGA CTG GGT TGG AAT AAG TTT GGA CAA GTA GG
Bottom 5’-CC TAC TTG TCC AAA CTT ATT CCA ACC CAG TCC ATA TAG TTT TCC ATC TG

W302F
Top 5’-GGA AAA CTA TAT GGA TGG GGT TTC AAT AAG TTT GGA CAA GTA GGA G
Bottom 5’-C TCC TAC TTG TCC AAA CTT ATT GAA ACC CCA TCC ATA TAG TTT TCC

W302L
Top 5’-GGA AAA CTA TAT GGA TGG GGT CTG AAT AAG TTT GGA CAA GTA GGA G
Bottom 5’-C TCC TAC TTG TCC AAA CTT ATT CAG ACC CCA TCC ATA TAG TTT TCC

W302H
Top 5’-GGA AAA CTA TAT GGA TGG GGT CAC AAT AAG TTT GGA CAA GTA GGA GTC G
Bottom 5’-C GAC TCC TAC TTG TCC AAA CTT ATT GTG ACC CCA TCC ATA TAG TTT TCC

W302Y
Top 5’-GGA AAA CTA TAT GGA TGG GGT TAC AAT AAG TTT GGA CAA GTA GGA G
Bottom 5’-C TCC TAC AAG TCC AAA CTT ATT GTA ACC CCA TCC ATA TAG TTT TCC

W337F
Top 5’-GAT GTT CAA GTC TCA TGT GGA TTC AGA CAT ACC TTG GCT GTC AC
Bottom 5’-GT GAC AGC CAA GGT ATG TCT GAA TCC ACA TGA GAC TTG AAC ATC

W352F
Top 5’-GAA AGA AAT AAC GTG TTT GCT TTC GGT AGA GGT ACA AAT GGA CAG
Bottom 5’-CTG TCC ATT TGT ACC TCT ACC GAA AGC AAA CAC GTT ATT TCT TTC

W352H
Top 5’-C GTG TTT GCT CAC GGT AGA GGT ACA AAT GGA CAG CTC GGC
Bottom 5’-GCC GAG CTG TCC ATT TGT ACC TCT ACC GTG AGC AAA CAC G

W352L
Top 5’-GA AAT AAC GTG TTT GCT CTG GGT AGA GGT ACA AAT GGA CAG CTC
Bottom 5’-GAG CTG TCC ATT TGT ACC TCT ACC CAG AGC AAA CAC GTT ATT TC

W400F
Top 5’-CCA TCT TCA GGG AAA AGC TTC GTG TCG CCT GCA GAG AG
Bottom 5’-GGA AAC TGA TAT TGT GTG CCG GAA TCC ACA AGC AAC CAT TG

R286A/W285F
Top 5’-C TCC CAG ATT TCG GGA GGT TTC GCA CAT ACA ATG GCA TTG ACT TCA G
Bottom 5’-C TGA AGT CAA TGC CAT TGT ATG TGC GAA ACC TCC CGA AAT CTG GGA G

R99A
Top 5’-GG GGA TGG GGT GAT TTT GGG GCA TTA GGC CAT GGT AAC TCA AGC G
Bottom 5’-C GCT TGA GTT ACC ATG GCC TAA TGC CCC AAA ATC ACC CCA TCC CC

D129N
Top 5’-C AAG CAG ATT GCT TGT GGG AAT AGT CAT TGT TTG GCT GTC
Bottom 5’-GAC AGC CAA ACA ATG ACT ATT CCC ACA AGC AAT CTG CTT G

D129K
Top 5’-C AAG CAG ATT GCT TGT GGG AAG AGT CAT TGT TTG GCT GTC
Bottom 5’-GAC AGC CAA ACA ATG ACT CTT CCC ACA AGC AAT CTG CTT G

R200A
Top 5’-C CTC TAT GGA TGG GGC TGG GGA GCA TAC GGA AAT TTG GGA TTA GG
Bottom 5’-CC TAA TCC CAA ATT TCC GTA TGC TCC CCA GCC CCA TCC ATA GAG G

R234Q
Top 5’-CA ATG GTT GCT TGT GGA TGG CAG CAC ACA ATA TCA GTT TCC
Bottom 5’-GGA AAC TGA TAT TGT GTG CTG CCA TCC ACA AGC AAC CAT TG

R234E
Top 5’-CA ATG GTT GCT TGT GGA TGG GAG CAC ACA ATA TCA GTT TCC
Bottom 5’-GGA AAC TGA TAT TGT GTG CTC CCA TCC ACA AGC AAC CAT TG

R286Q
Top 5’-CC CAG ATT TCG GGA GGT TGG CAA CAT ACA ATG GCA TTG AC-3’
Bottom 5’--GT CAA TGC CAT TGT ATG TTG CCA ACC TCC CGA AAT CTG GG-3’

R338Q

Top 5'-CAA GTC TCA TGT GGA TGG CAA CAT ACC TTG GCT GTC ACT G
Bottom 5'-C AGT GAC AGC CAA GGT ATG TTG CCA TCC ACA TGA GAC TTG

R354A
Top 5'-C GTG TTT GCT TGG GGT GCA GGT ACA AAT GGA CAG CTC GGC
Bottom 5'-GCC GAG CTG TCC ATT TGT ACC TGC ACC CCA AGC AAA CAC G

**Primer sequences (UVR8 deltaC).**
The three-letter codon corresponding to the mutated amino acid residue(s) is highlighted in red. For each primer pair, the sequence is shown from 5' to 3'.

W94G
Top 5'-C ATG GAA GTC TAC AGT TGG GGA GGG GGT GAT TTT GGG AGA TTA GG
Bottom 5'-CC TAA TCT CCC AAA ATC ACC CCC TCC CCA ACT GTA GAC TTC CAT G

W233G
Top 5'-G TCA ATG GTT GCT TGT GGA GGG CGG CAC ACA ATA TCA GTT TCC
Bottom 5'-GGA AAC TGA TAT TGT GTG CCG CCC TCC ACA AGC AAC CAT TGA C

W233K
Top 5'-G TCA ATG GTT GCT TGT GGA AAG CGG CAC ACA ATA TCA GTT TCC
Bottom 5'-GGA AAC TGA TAT TGT GTG CCG CTT TCC ACA AGC AAC CAT TGA C

W233D
Top 5'-G TCA ATG GTT GCT TGT GGA GAC CGG CAC ACA ATA TCA GTT TCC
Bottom 5'-GGA AAC TGA TAT TGT GTG CCG GTC TCC ACA AGC AAC CAT TGA C

W285G
Top 5'-CC CAG ATT TCG GGA GGT **GGG** AGA CAT ACA ATG GCA TTG AC
Bottom 5'-GT CAA TGC CAT TGT ATG TCT CCC ACC TCC CGA AAT CTG GG

W285K
Top 5'-C TCC CAG ATT TCG GGA GGT **AAG** AGA CAT ACA ATG GCA TTG ACT TC
Bottom 5'-GA AGT CAA TGC CAT TGT ATG TCT CTT ACC TCC CGA AAT CTG GGA G

W285D
Top 5'-C TCC CAG ATT TCG GGA GGT **GAC** AGA CAT ACA ATG GCA TTG ACT TC
Bottom 5'-GA AGT CAA TGC CAT TGT ATG TCT GTC ACC TCC CGA AAT CTG GGA G

W337G
Top 5'-GTA GTT CAA GTC TCA TGT GGA **GGG** AGA CAT ACC TTG GCT GTC ACTG
Bottom 5'-C AGT GAC AGC CAA GGT ATG TCT CCC TCC ACA TGA GAC TTG AAC TAC

W337K

Top 5’-GTA GTT CAA GTC TCA TGT GGA **AAG** AGA CAT ACC TTG GCT GTC ACT G
Bottom 5’-C AGT GAC AGC CAA GGT ATG TCT CTT TCC ACA TGA GAC TTG AAC TAC

W337D
Top 5’-GTA GTT CAA GTC TCA TGT GGA **GAC** AGA CAT ACC TTG GCT GTC ACT G
Bottom 5’-C AGT GAC AGC CAA GGT ATG TCT GTC TCC ACA TGA GAC TTG AAC TAC

D96N
Top 5’-C TAC AGT TGG GGA TGG GGT AAT TTT GGG AGA TTA GGC CAT GG
Bottom 5’-CC ATG GCC TAA TCT CCC AAA ATT ACC CCA TCC CCA ACT GTA G

D107N
Top 5’-GGC CAT GGT AAC TCA AGC AAC TTG TTT ACT CCG CTA CC
Bottom 5’-GG TAG CGG AGT AAA CAA GTT GCT TGA GTT ACC ATG GCC

R286D
Top 5’-CC CAG ATT TCG GGA GGT TGG GAC CAT ACA ATG GCA TTG ACT TC
Bottom 5’-GA AGT CAA TGC CAT TGT ATG GTC CCA ACC TCC CGA AAT CTG GG

D96R
Top 5’-C TAC AGT TGG GGA TGG GGT AGG TTT GGG AGA TTA GGC CAT GG
Bottom 5’-CC ATG GCC TAA TCT CCC AAA CCT ACC CCA TCC CCA ACT GTA G

D107R
Top 5’-GGC CAT GGT AAC TCA AGC AGG TTG TTT ACT CCG CTA CCA ATC
Bottom 5’-GAT TGG TAG CGG AGT AAA CAA CCT GCT TGA GTT ACC ATG GCC

R338D
Top 5’-GTT CAA GTC TCA TGT GGA TGG GAC CAT ACC TTG GCT GTC ACT G
Bottom 5’-C AGT GAC AGC CAA GGT ATG GTC CCA TCC ACA TGA GAC TTG AAC

E43R/D44R
Top 5’-GT TCT TGG GGT CGA GGA AGG AGG GGA CAG TTA GGT CAT GGC GAT GC
Bottom 5’-GC ATC GCC ATG ACC TAA CTG TCC CCT CCT TCC TCG ACC CCA AGA AC

E43Q/D44R
Top 5’-GT TCT TGG GGT CGA GGA CAG AGG GGA CAG TTA GGT CAT GGC G
Bottom 5’-C GCC ATG ACC TAA CTG TCC CCT CTG TCC TCG ACC CCA AGA AC

E43R/D44N
Top 5’-GT TCT TGG GGT CGA GGA AGG AAT GGA CAG TTA GGT CAT GGC G
Bottom 5’-C GCC ATG ACC TAA CTG TCC ATT CCT TCC TCG ACC CCA AGA AC

**Kinetic model fitting for transient absorption data at 315-nm excitation and $E_{ET}$ calculation.**

The 315-nm excitation light selectively excites the pyramid center tryptophan ($4W_c$).[1] $4W_c$ have reactive (undergo W285-W233 charge separation) and nonreactive (no charge separation) subpopulations. The reactive subpopulation undergoes reactions as schemed in Fig. 2f. The nonreactive species simply relaxes to the ground state with the time constant $\tau_{c2}$ (1.4 ns). Assuming the total tryptophan excited state concentration is $n_0$ upon excitation at time zero with the reactive fraction $R_{c1}$ (0.75, Fig. 2b), the temporal evolution of various intermediates can be solved by the following equations:

Reactive W*decays with time constant $\tau_{cs}$ (80 ps, Fig. 2b):

$$[W_{c1}]_t = n_0 R_{c1} e^{-\frac{t}{\tau_{CS}}} \tag{1}$$

Nonreactive W* decays with time constant $\tau_{c2}$ (1.4 ns, Fig. 2b):

$$[W_{c2}]_t = n_0(1-R_{c1}) e^{-\frac{t}{\tau_{c2}}} \tag{2}$$

Charge separated state is formed via CS reaction and decays by parallel CR, ET and DP channels:

$$\frac{d[W285^{-}W233^{+}]_t}{dt} = \tau_{CS}{}^{-1}[W_{c1}]_t - (\tau_{CR}{}^{-1} + \tau_{ET}{}^{-1} + \tau_{DP}{}^{-1})[W285^{-}W233^{+}]_t \tag{3}$$

W285 neutral W233 plus forms by ET channel and decays in DP channel:

$$\frac{d[W285W233^{+}]_t}{dt} = \tau_{ET}{}^{-1}[W285^{-}W233^{+}]_t - \tau_{DP}{}^{-1}[W285W233^{+}]_t \tag{4}$$

W285 minus W233 neutral radical is formed by DP and decays in ET to Arg:

$$\frac{d[W285^{-}W233^{\bullet}]_t}{dt} = \tau_{DP}{}^{-1}[W285^{-}W233^{+}]_t - \tau_{ET}{}^{-1}[W285^{-}W233^{\bullet}]_t \tag{5}$$

where $\tau_{CS}$, $\tau_{c2}$, $\tau_{CR}$, $\tau_{ET}$ and $\tau_{DP}$ are time scales of charge separation (CS), lifetime decay of the nonreactive population, charge recombination (CR), electron transfer to arginine from W285 anionic radicals (ET) and deprotonation of $W233^{+\bullet}$ (DP), respectively. By solving all differential equations, we found that:

$$[W285^{-}W233^{+}]_t = \frac{n_0 R_{c1}\tau_{CS}{}^{-1}}{\tau_{CR}{}^{-1} + \tau_{ET}{}^{-1} + \tau_{DP}{}^{-1} - \tau_{CS}{}^{-1}}[e^{-\tau_{CS}{}^{-1}t} - e^{-(\tau_{CR}{}^{-1}+\tau_{ET}{}^{-1}+\tau_{DP}{}^{-1})t}] \tag{6}$$

$$\begin{aligned}[W285W233^{+}]_t = \frac{n_0 R_{c1}\tau_{CS}{}^{-1}\tau_{ET}{}^{-1}}{\tau_{CR}{}^{-1} + \tau_{ET}{}^{-1} + \tau_{DP}{}^{-1} - \tau_{CS}{}^{-1}}&[(\frac{1}{\tau_{CS}{}^{-1} - \tau_{DP}{}^{-1}} - \frac{1}{\tau_{CR}{}^{-1} + \tau_{ET}{}^{-1}})e^{-\tau_{DP}{}^{-1}t} \\ &- \frac{1}{\tau_{CS}{}^{-1} - \tau_{DP}{}^{-1}} e^{-\tau_{CS}{}^{-1}t} + \frac{1}{\tau_{CR}{}^{-1} + \tau_{ET}{}^{-1}} e^{-(\tau_{CR}{}^{-1}+\tau_{ET}{}^{-1}+\tau_{DP}{}^{-1})t}]\end{aligned} \tag{7}$$

$$\begin{aligned}[W285^{-}W233^{\bullet}]_t = \frac{n_0 R_{c1}\tau_{CS}{}^{-1}\tau_{DP}{}^{-1}}{\tau_{CR}{}^{-1} + \tau_{ET}{}^{-1} + \tau_{DP}{}^{-1} - \tau_{CS}{}^{-1}}&[(\frac{1}{\tau_{CS}{}^{-1} - \tau_{ET}{}^{-1}} - \frac{1}{\tau_{CR}{}^{-1} + \tau_{DP}{}^{-1}})e^{-\tau_{ET}{}^{-1}t} \\ &- \frac{1}{\tau_{CS}{}^{-1} - \tau_{ET}{}^{-1}} e^{-\tau_{CS}{}^{-1}t} + \frac{1}{\tau_{CR}{}^{-1} + \tau_{DP}{}^{-1}} e^{-(\tau_{CR}{}^{-1}+\tau_{ET}{}^{-1}+\tau_{DP}{}^{-1})t}]\end{aligned} \tag{8}$$

$$[W^{+}]_t = [W285^{-}W233^{+}] + [W285W233^{+}] = \frac{n_0 R_{c1}\tau_{CS}{}^{-1}}{\tau_{CR}{}^{-1} + \tau_{ET}{}^{-1} + \tau_{DP}{}^{-1} - \tau_{CS}{}^{-1}}[(\frac{\tau_{ET}{}^{-1}}{\tau_{CS}{}^{-1} - \tau_{DP}{}^{-1}} - \frac{\tau_{ET}{}^{-1}}{\tau_{CR}{}^{-1} + \tau_{ET}{}^{-1}})e^{-\tau_{DP}{}^{-1}t}$$

$$+(1-\frac{\tau_{ET}^{-1}}{\tau_{CS}^{-1}-\tau_{DP}^{-1}})e^{-\tau_{CS}^{-1}t}-(1-\frac{\tau_{ET}^{-1}}{\tau_{CR}^{-1}+\tau_{ET}^{-1}})e^{-(\tau_{CR}^{-1}+\tau_{ET}^{-1}+\tau_{DP}^{-1})t}] \quad (9)$$

Total W* population:

$$[W^*]_t=[W_{c1}]_t+[W_{c2}]_t=n_0R_{c1}e^{-\tau_{CS}^{-1}t}+n_0(1-R_{c1})e^{-\tau_{c2}^{-1}t} \quad (10)$$

By fitting the $W^{+\bullet}$ signal (green dashed line in Fig. 2e) using equation 9, $\tau_{ET}$, $\tau_{CR}$ and $\tau_{DP}$ were obtained as 17 ps, 4 ps and 3 ns. Due to relatively slow CS compared to ET and CR steps, from equation 6, $W285^{-\bullet}W233^{+\bullet}$ shows a rise time scale corresponding to total rate of CR, ET and DP (~3.2 ps, equation 11); a decay time of $\tau_{CS}$ in 80 ps. By equation 7, $W285W233^{+\bullet}$ has a rise time of $\tau_{CS}$ (80 ps); a decay time of $\tau_{DP}$ (3 ns as determined below); a decay component with the total time scale of CR, ET and DP (~3.2 ps). By summing the temporal evolution of the two species (equation 9), $W^{+\bullet}$ signal have two rise components: 80 ps and ~3.2 ps; and a decay time of $\tau_{DP}$.

$$\tau_{CR}^{-1}+\tau_{ET}^{-1}+\tau_{DP}^{-1}=(4ps)^{-1}+(17ps)^{-1}+(3000ps)^{-1}=(3.2ps)^{-1} \quad (11)$$

Thus, branching ratio of ET ($E_{ET}$) was calculated with $\tau_{CR}$, $\tau_{ET}$ and $\tau_{DP}$ which are 4 ps, 17 ps and 3 ns, respectively (equation 12):

$$E_{ET}=\frac{(\tau_{ET})^{-1}+(\tau_{DP})^{-1}}{(\tau_{ET})^{-1}+(\tau_{CR})^{-1}+(\tau_{DP})^{-1}}=\frac{(17ps)^{-1}+(3000ps)^{-1}}{(17ps)^{-1}+(4ps)^{-1}+(3000ps)^{-1}}=0.19 \quad (12)$$

According to the Beer-Lambert law, the signal of absorption transient at 630 nm probe wavelength can be acquired as:

$$\Delta A\propto \varepsilon_{W*}[W^*]_t+\varepsilon_{W^+}[W^+]_t \quad (13)$$

By model fitting, extinction coefficients of W* and $W^{+\bullet}$ have a ratio of 4:3, consistent with previous studies by Kohler et al.[2] determining the extinction coefficients of W* ($\varepsilon_{W*}$) and $W^{+\bullet}$ ($\varepsilon_{W+}$) of 4000 $M^{-1}cm^{-1}$ and 3000 $M^{-1}cm^{-1}$, respectively.

**Numerical simulation of absorption transients at 290 nm excitation.**

290-nm light can pump all 3 Trp groups: distal tryptophan ($6W_d$), peripheral tryptophan ($3W_p$) and pyramid center tryptophan ($4W_c$). We first simulated W excited state (W*) temporal evolution for 3 groups with the energy transfer model as described in another study[1]. With $[W_{c1}]_t$, population evolution of electron transfer intermediates was simulated using equations 6-9.

Total W* population of all 3 Trp groups:

$$[W^*]_t=[W_{c1}]_t+[W_{c2}]_t+[W_p]_t+[W_d]_t \quad (14)$$

The signal of absorption transient at 630 nm probe wavelength, and signal contributions from W* and $W^{+\bullet}$ can be acquired as:

$$\Delta A\propto \varepsilon_{W*}[W^*]_t+\varepsilon_{W^+}[W^+]_t=S(W^*)+S(W^+) \quad (15)$$

In our simulations, the extinction coefficients of W* ($\varepsilon_{W*}$) and $W^{+\bullet}$ ($\varepsilon_{W+}$) were set to 4000 $M^{-1}cm^{-1}$ and 3000 $M^{-1}cm^{-1}$, respectively[2]. $R_{c1}$, $\tau_{CS}$ and $\tau_{c2}$ were 0.75, 80 ps and 1.4 ns respectively. $\tau_{CR}$, $\tau_{ET}$ and $\tau_{DP}$ values were 4 ps, 17 ps and 3 ns respectively. The simulated results are shown in Supplementary Fig. 4.

**QM/MM calculations.**

The side chains of residues in QM region were terminated between $C_\beta$ and $C_\alpha$ atoms of with a hydrogen link atom. The corresponding backbone and other residues were treated as MM region. We extracted 100 snapshots (one snapshot every 200 ps) from the 20 ns production simulations of the parent state, electronic excited states and charge transfer states to statistically investigate the electron transfer (ET) rate based on the QM/MM calculations. The single excited state of residue was acquired by time dependent range-separated hybrid functional TD-CAM-B3LYP.[3] MSDFT calculations were performed by PBE0 functional[4] with HF correction for the off-diagonal Hamiltonian matrix element. 6-31+G(d) basis set was used for all the calculation. All the QM/MM calculations were performed with a locally modified version of GAMESS code[5] in CHARMM quantum part.

For QM/MM calculation, the diabatic states of electronic localized excitation and electron transfer can be written as[6]:

$$\Phi_{ab}(S_1) = \underbrace{\Psi_1 \cdots \hat{A}\{\Psi_a^{S_1}\Psi_b^{S_0}\}\cdots\Psi_N}_{N-1\ \text{fragments}} \tag{16}$$

$$\Phi_{ab}(CT) = \underbrace{\Psi_1 \cdots \hat{A}\{\Psi_a^{+\bullet}\Psi_b^{-\bullet}\}\cdots\Psi_N}_{N-1\ \text{fragments}} \tag{17}$$

where $\hat{A}\{\Psi_a^{S_1}\Psi_b^{S_0}\}$ specifies an antisymmetric wave function for the locally excited residue *a*, coupled with residue *b* in the ground state, while $\hat{A}\{\Psi_a^{+\bullet}\Psi_b^{-\bullet}\}$ is an antisymmetric wave function for electron transfer from residue a to residue b. They can be constructed using the corresponding fragment block-localized KS orbitals. The other N-1 fragments are treated as classically molecular mechanics (MM). The QM part includes side chains of W285/W233, W285/W233/D129, W285/R286/D97/D107 and W285/R338/E43/D44 for calculating W285/W233 charge separation, charge recombination, ET to R286 and ET to R338, respectively. The charged residue of E43, D44, D97, D107, and D129 were treated as QM region because they are very close to the key residue of W233, W285, R286 and R338 in active sites, which has a significant quantum effect. MSDFT was used to construct the diabatic states of electronic localized excitation and electron transfer. TD-CAM-B3LYP together with MSDFT was used to obtain the locally excited W233. In addition, MSDFT was used to calculate electronic coupling matrix element between two diabatic states for rate constants.

Using Marcus-Hush theory[7,8] under the harmonic approximation, two parabolas along a reaction coordinate can be used to represent the potential energy curves of reaction and product states. In the diabatic representation, the two curves cross and the crossing point is the transition state. With the assumption that both parabolas have the same curvature, the activation energy $\Delta G^+$ is given by

$$\Delta G^+ = \frac{(\lambda + \Delta G^0)^2}{4\lambda}$$

(18)

here $\Delta G^0$ is the driving force and λ is the reorganization energy owing to the geometric relaxation accompanying. For the nonadiabatic ET, the rate constant $k_{\mathrm{ET}}$ is calculated by taking golden rule-based expression in the high temperature limit, i.e.,[9,10]

$$k_{\mathrm{ET}} = \frac{4\pi}{h} V_{\mathrm{rp}}^2 \left( \frac{1}{4\pi\lambda k_{\mathrm{B}} T} \right)^{1/2} \exp\left[ -\left(\lambda + \Delta G^0\right)^2 \Big/ 4\lambda k_{\mathrm{B}} T \right] \tag{19}$$

where $h$ and $k_B$ represent Plank and Boltzmann constant, respectively, and $T$ stands for the temperature. $V_{rp}$ is the electronic coupling matrix element, which is half of energy gap formed an upper and a lower curve at the crossing point. In the adiabatic representation that the two curves avoid crossing. The reorganization energy $\lambda$ and driving force $\Delta G^0$ can be obtained using linear response method[11] (see Supplementary Scheme 1):

$$\lambda = \frac{1}{2}[\left(G(\mathrm{P})_r - G(\mathrm{P})_\mathrm{p}\right) + (G(\mathrm{R})_p - G(\mathrm{R})_r]$$
$$= \frac{1}{2}[(G(\mathrm{P}) - G(\mathrm{R}))_r + (G(\mathrm{R}) - G(\mathrm{P}))_p]$$

(20)

$$\Delta G^o = \frac{1}{2}[\left(G(\mathrm{P})_r - G(\mathrm{R})_p\right) + (G(\mathrm{P})_p - G(\mathrm{R})_r]$$
$$= \frac{1}{2}[(G(\mathrm{P}) - G(\mathrm{R}))_r - (G(\mathrm{R}) - G(\mathrm{P}))_p]$$

(21)

here capital P and R stand for product and reactant electronic states while lowercase p and r means the equilibrium structures of product and reactant states. The free energy of these four points can be statistically calculated from 100 selected snapshots using QM/MM methods based on block localized density functional theory (BLDFT).

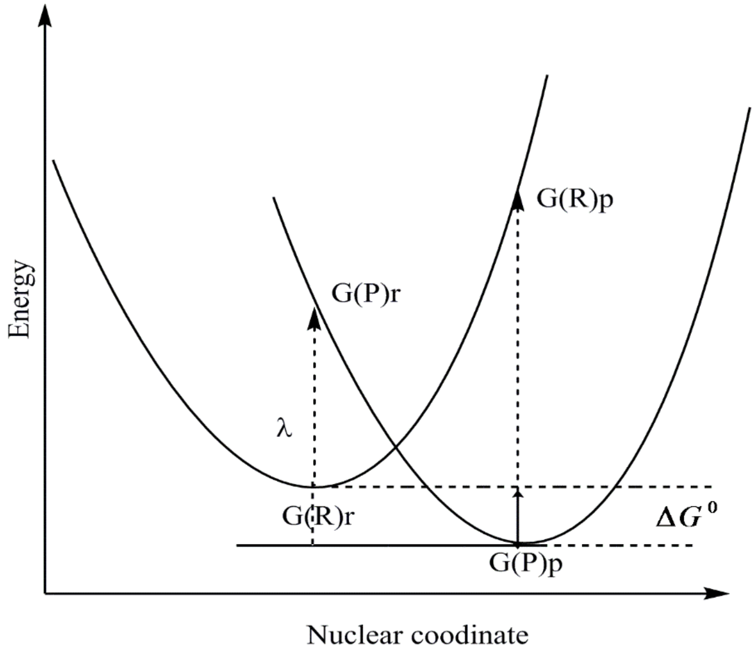


**Supplementary Scheme 1 | Reorganization energy and driving force using linear response method.**

The coupling energies are calculated as $V_{rp} = |\langle \Phi_{ab}(r) | H | \Phi_{ab}(p) \rangle - S_{rp}\varepsilon_g|$ where $S_{rp}$ is the overlap integral between the two nonorthogonal Kohn-Sham determinants for state r and p, and

$\varepsilon_g$ is the adiabatic parent state energy obtained as lowest root by diagonalizing the 2×2 Hamiltonian matrix. The first term is off-diagonal Hamiltonian matrix element: $H_{rp} = |\langle \Phi_{ab}(r) | H | \Phi_{ab}(p) \rangle$ which has both the implicit functional of the diabatic state density and the transition density. It can be calculated by[12,13]:

$$H_{rp} = H_{rp}^{\mathrm{BLKS}} + S_{rp} \frac{\Delta E_r^c + \Delta E_p^c}{2} \tag{22}$$

where $\Delta E_r^c$ and $\Delta E_p^c$ are correlation energy for two diabatic states, which can be approximated by the energy difference between BLDFT and HF theory using BLKS orbitals. Results are shown in Supplementary Fig. 14 and Supplementary Table 3.

**Supplementary Text**

The fluorescence quantum yields ($Q_F$) of UVR8 WT and W285F are 0.07 and 0.42, respectively. If there is no energy transfer from distal or peripheral tryptophan residues to the pyramid center, we can set the average fluorescence QY of 3 $W_c$ (W94, W233 and W337) as X in W285F and set the average fluorescence QY of the other 10 tryptophan residues as Y. In W285F, the overall fluorescence QY is the weighted average of all 13 tryptophan residues: (3*X+10*Y)/13=0.42. In WT, 10*Y/14<0.07. Thus, 3*X>13*0.42-14*0.07=4.48, X>1.49. Fluorescence QY should not exceed unity. There must be energy transfer from distal/peripheral tryptophan residues to the pyramid center.

## Supplementary Tables

**Supplementary Table 1 | UVR8 tryptophan (W) mutant summary.**

| Mutant | Yield | Dimer/Monomer | UV Response | Emission Peak |
|---|---|---|---|---|
| WT | normal | Dimer | + | 332 nm |
| W285F | normal | Dimer | - | 356 nm |
| W233F | normal | Dimer | - | 335 nm |
| W94F | normal | Dimer | + | 332 nm |
| W337F | normal | Dimer | + | 333 nm |
| W39F | normal | Dimer | + | 336 nm |
| W92F | normal | Dimer | + | 333 nm |
| W144F | normal | Dimer | + | 333 nm |
| W196F | low | Dimer | + | 336 nm |
| W198F | normal | Dimer | + | 332 nm |
| W250F | normal | Dimer | + | 334 nm |
| W300F | very low | Dimer | + | 335 nm |
| W302F | very low | Dimer | + | 334 nm |
| W352F | no yield | | | |
| W400F | normal | Dimer | + | 334 nm |
| W285/233F | high | Dimer | - | 335 nm |
| W285/94F | normal | Dimer | - | 353 nm |
| W285/337F | normal | Dimer | - | 354 nm |
| W233/94F | normal | Dimer | - | 335 nm |
| W233/337F | normal | Dimer | - | 337 nm |
| W94/337F | normal | Dimer | + | 334 nm |
| W285/233/94F | normal | Dimer | - | 336 nm |
| W285/94/337F | normal | Dimer | - | 349 nm |
| W233/94/337F | normal | Dimer | - | 335 nm |
| W285/233/337F | normal | Dimer | - | 336 nm |
| W285/233/94/337F | normal | Dimer | - | 335 nm |
| W285/233/94/337/250F | normal | Dimer | - | 335 nm |
| W285/233/94/337/198/250F (6WF) | normal | Dimer | - | 331 nm |
| W285/233/94/337/198/250/302F | no yield | | | |
| W285/300F | tiny | Dimer | - | |
| W285/300/39/196F | | | | |
| W285/302F | tiny | Dimer | - | |
| W285/300/39F | low | Dimer | - | 355 nm |
| W285/302/250F | tiny | Dimer | - | 355 nm |
| W300/39F | very low | Dimer | + | 336 nm |
| W300/39/196F | very low | Dimer | + | 338 nm |
| W285/233/302/250F | tiny | | | |
| W285Y | normal | Dimer | - | 354 nm |
| W233Y | normal | Dimer | - | 334 nm |
| W285/233Y | high | Dimer | - | 335 nm |
| W285F D129N | normal | Dimer | - | 340 nm |
| W39/92F | normal | Dimer | + | |
| W39/92/144F | normal | Dimer | + | 337 nm |

**Supplementary Table 1 | UVR8 tryptophan (W) mutant summary. (continued)**

| Mutant | Yield | Dimer/Monomer | UV Response | Emission Peak |
|---|---|---|---|---|
| W39/92/144/196F | very low | Dimer | + | 338 nm |
| W39/196/300/352F | no yield | | | |
| W39/196/300/352/92F | no yield | | | |
| W94/233/285/337/250/302F | no yield | | | |
| W39/92/196/300/352F | no yield | | | |
| W39/196/300/352/92/144F | no yield | | | |
| W250/302F | very low | Dimer | + | 335 nm |
| W285F D129K | low | Dimer | - | 338 nm |
| W250/302F | very low | Dimer | + | 335 nm |
| W285F D129K | low | Dimer | - | 338 nm |
| W250/198F | normal | Dimer | + | 331 nm |
| W300H | no yield | | | |
| W300L | no yield | | | |
| W302H | low | Dimer | + | 334 nm |
| W302L | no yield | | | |
| W302Y | low | Dimer | + | 334 nm |
| W39/92/198/144F W352Y | no yield | | | |
| W352H | tiny | | | |
| W352L | no yield | | | |
| W39/92/144/198F | normal | | | |
| W39/92/144/198/250F | very low | Dimer | + | |
| W39/92/144/198/250/400F | tiny | Dimer | - | 338 nm |
| W285/233/94/337/198F | normal | Dimer | - | |
| W94/198/250F | very low | Dimer | weak | 335 nm |
| W94/198F | low | | | |
| W92/196F | low | | | |
| W285A | normal | Dimer | - | 348 nm |
| W94/233/285/337/400F | normal | Dimer | - | 335 nm |
| W94/233/285/337/198/250/400F | normal | Dimer | - | 331 nm |
| W94/233/285/337/198/250/400F W302A | no yield | | | |
| W94/233/285/337/198/250/400F W302S | no yield | | | |
| W94/233/285/337/198/250/400F W302D | no yield | | | |
| W94/233/285/337/198/250/400F W302K | no yield | | | |
| W94/233/285/337/198/250/400F W302Y | no yield | | | |
| W94/233/285/337/198/250/400F W302H | no yield | | | |
| W94/233/285/337/198/250/400F W302G | no yield | | | |

**Supplementary Table 1 | UVR8 tryptophan (W) mutant summary. (continued)**

| Mutant | Yield | Dimer/Monomer | UV Response | Emission Peak |
|---|---|---|---|---|
| W94/233/285/337/198/250/400F W302T | no yield | | | |
| W94/233/285/337/198/250/400F W302I | no yield | | | |
| W94/233/285/337/198/250/400F W302E | no yield | | | |
| W94/233/285/337/198/250/400F W302R | no yield | | | |
| W94/233/285/337/198/250/400F W302P | no yield | | | |
| W94/233/285/337/198/250/400F W302N | no yield | | | |
| W94/233/285/337/198/250/400F W302V | no yield | | | |
| W94/233/285/337/198/250/400F W302Q | no yield | | | |
| W94/233/285/337/198/250/400F W302C | no yield | | | |
| W94/233/285/337/198/250/400F W302M | no yield | | | |
| W94/233/285/337/198/250/400F R286A W302H (Mono-6$W_d$) | normal | Monomer | | 320 nm |
| W94/233/285/337/198/250/400F R338A W302H | normal | Monomer | | 320 nm |
| ΔC mutants | | | | |
| W285G | normal | Dimer | - | 346 |
| W285D | normal | Dimer | - | 346 |
| W285K | no yield | Dimer | - | |
| W233G | normal | Dimer | - | 333 |
| W94G | normal | Dimer | + | 331 |
| W337D | normal | Dimer | + | |
| W337G | normal | Dimer | + | 336 |
| W337K | no yield | | | |
| W285/233/94/337G | high | Dimer | - | 334 |
| W285K/W233D | high | Dimer | - | 334 |
| W285D/W233K | high | Dimer | - | 335 |
| W285/233/94/337/198/250F | no yield | | | |
| W285/233/94/337/198/250/302F | no yield | | | |

All protein yields are the yields relative to WT (0.2 mg per liter of cell culture). Emission peaks have ±1 nm experimental error.

**Supplementary Table 2 | UVR8 charged residue mutant summary.**

| Mutant | Yield | Dimer/Monomer | UV Response | Emission Peak |
|---|---|---|---|---|
| D129K | low | Dimer | weak | 334 nm |
| R146A | low | Dimer | + | 333 nm |
| R200A | normal | Dimer | + | 336 nm |
| D129N | normal | Dimer | + | 334 nm |
| R286A | normal | Monomer | | 340 nm |
| R286Q | normal | Monomer | | 342 nm |
| R286K | normal | Dimer | + | 336 nm |
| R338A | normal | Monomer | | 341 nm |
| R99A | normal | Dimer | + | 336 nm |
| R338Q | normal | Monomer | | 341 nm |
| R338K | normal | Dimer | + | 337 nm |
| R234Q | low | Dimer | + | 336 nm |
| R146/354A | no yield | | | |
| R146/200A | low | Dimer | + | 337 nm |
| ΔC mutants | | | | |
| D96N | low | Dimer | + | 337 nm |
| D107N | normal | Dimer | + | 336 nm |
| D96/107N | low | most Monomer | | 338 nm |
| R234E | tiny | Dimer | | |
| D96R | no yield | | | |
| E43Q/D44R | no yield | | | |
| E43R/D44N | no yield | | | |
| R286D/D96R | no yield | | | |
| E43R/D44N/R338D | normal | Monomer | | |
| R286D/D96R/D107N | low | Monomer | | |
| R286D/D96R/D107R | no yield | | | |
| R286D/D96N/D107R | no yield | | | |

All protein yields are the yields relative to WT (0.2 mg per liter of cell culture). Emission peaks have ±1 nm experimental error.

**Supplementary Table 3 | QM/MM computed rate constants for the initial electron transfer reactions from the photochemically excited W233* to W285, $W233^{+\bullet}$ $W285^{-\bullet}$ change recombination, and the subsequent charge propagation to neutralize either R286 or R338.**

| | $V_{12}$ (meV) | $\Delta G^0$ (kcal/mol) | $\lambda$ (kcal/mol) | $\Delta G^+$ (kcal/mol) | $k_{ET}$ ($s^{-1}$) |
|---|---|---|---|---|---|
| $W233^{*}W285 \rightarrow W233^{+\bullet}W285^{-\bullet}$ | 37.7 | -0.3 | 18.6 | 4.5 | $1.4 \times 10^{10}$ |
| $W233^{+\bullet}W285^{-\bullet} \rightarrow W233W285$ | 23.2 | -0.2 | 5.3 | 1.2 | $2.4 \times 10^{12}$ |
| $W285^{-\bullet}R286^{+} \rightarrow W285R286^{\bullet}$ | 52.7 | -3.5 | 17.5 | 2.8 | $4.8 \times 10^{11}$ |
| $W285^{-\bullet}R338^{+} \rightarrow W285R338^{\bullet}$ | 61.6 | -3.9 | 18.5 | 2.9 | $5.6 \times 10^{11}$ |

The latter residues are located nearly symmetrically on both sides of W285 and have similar rates for electron transfer as acceptors from the transient intermediate $W285^{-\bullet}$ anionic radical. $V_{12}$, $\Delta G^0$, $\lambda$, $\Delta G^+$, $k_{ET}$ are the calculated electronic couplings, driving forces, reorganization energy, free energy barriers and electron transfer rates.

**Supplementary Figures**

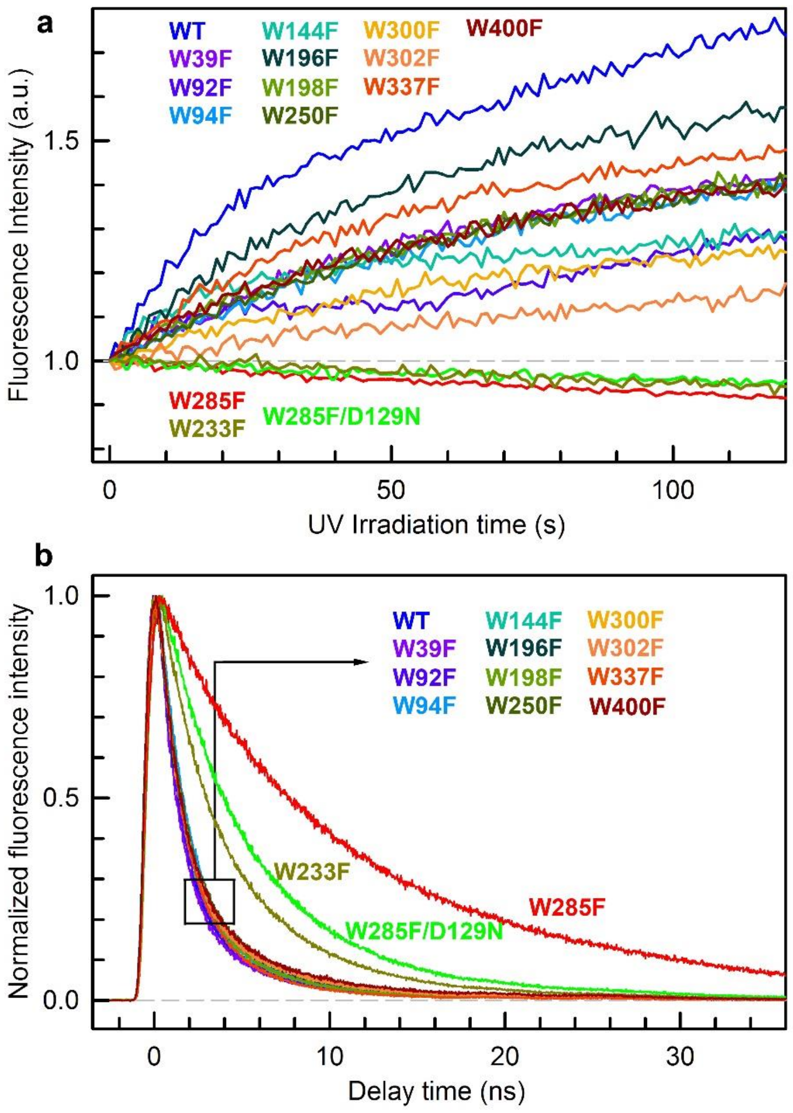


**Supplementary Fig. 1 | UV-induced dissociation function test and time-resolved fluorescence of single-point tryptophan mutants. a**, Fluorescence intensity (290-nm excitation) changes under continuous UV illumination for WT, 13 single-point tryptophan-to-phenylalanine mutants (except W352) and W285F/D129N. An increasing fluorescence intensity indicates normal dissociation function, whereas a decreasing peak intensity is due to photobleaching of the nonfunctional mutants. **b**, Time-resolved fluorescence transients of WT, 13 single-point tryptophan mutants and W285F/D129N are determined by sub-nanosecond TCSPC. Note that all mutants, except W233F, W285F and W285F/D129N, have similar fluorescence decay transients.

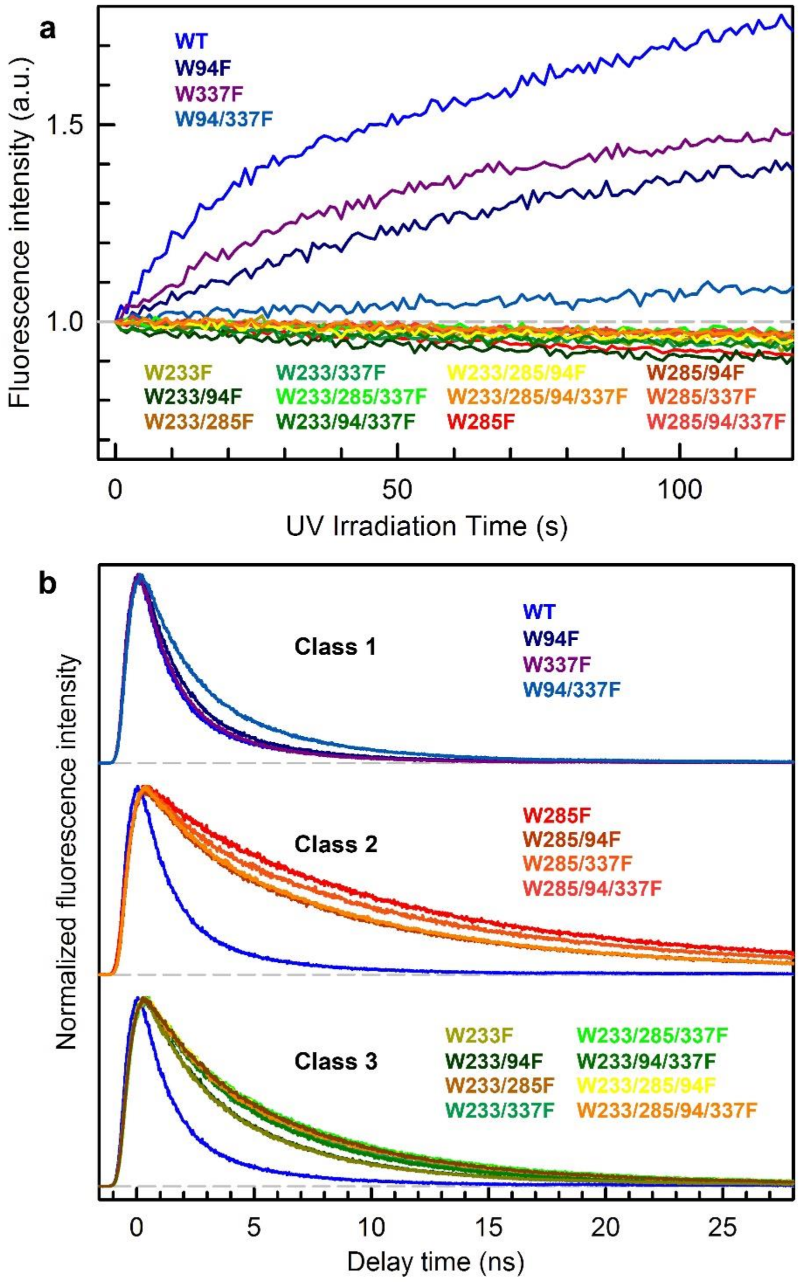


**Supplementary Fig. 2 | UV-induced dissociation function test and time-resolved fluorescence of tryptophan pyramid center mutants. a**, Fluorescence intensity (290-nm excitation) changes under continuous UV illumination for WT and 15 tryptophan-to-phenylalanine mutants. An increasing fluorescence intensity indicates normal dissociation function, whereas a decreasing intensity is due to photobleaching of the nonfunctional mutants. **b**, Time-resolved fluorescence transients of WT and 15 combinations of tryptophan-to-phenylalanine mutants are determined by sub-nanosecond TCSPC. According to their emission spectra and function, we categorized these mutants into three classes.

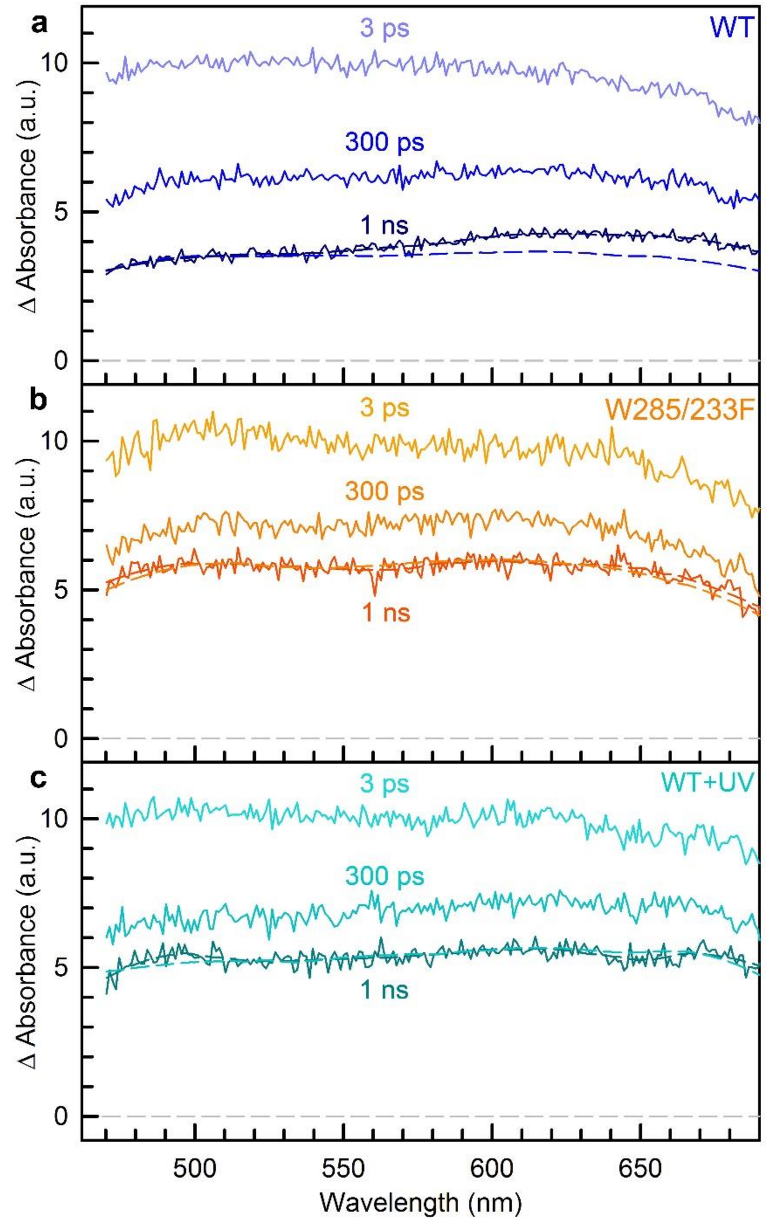


**Supplementary Fig. 3 | Femtosecond-resolved broadband absorption spectra (290-nm excitation) of various samples. a**, WT dimer. **b**, W285/233F. **c**, WT after UV-B irradiation (WT monomer). Dashed lines are renormalized 3-ps and 300-ps spectra for comparison with 1-ns spectra. Note that 300-ps and 1-ns spectra of W285/233F and WT+UV samples are very close (dashed orange and cyan lines). However, a significant difference was observed in WT dimer which is due to formation of $W^{+•}$ intermediate.

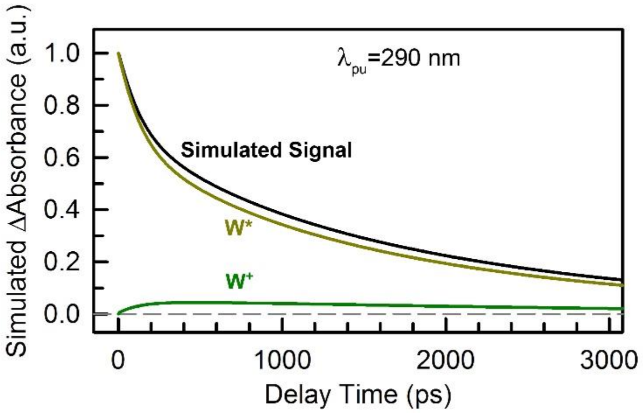


**Supplementary Fig. 4 | Trp excited state signal dominates at 290-nm excitation.** Numerical simulations of signal ratios of Trp excited states (W*) and Trp cationic radicals ($W^{+•}$) in WT (290-nm excitation). The initial excited populations and energy transfer rates were based on our proposed energy transfer models[1]. In the simulations, charge separation time is 80 ps, charge recombination time is 4 ps, ET to Arg is 17 ps and deprotonation time is 3 ns. Extinction coefficients of W* and $W^{+•}$ were set to 4000 $M^{-1}cm^{-1}$ and 3000 $M^{-1}cm^{-1}$ respectively, according to Kohler et al.[2]

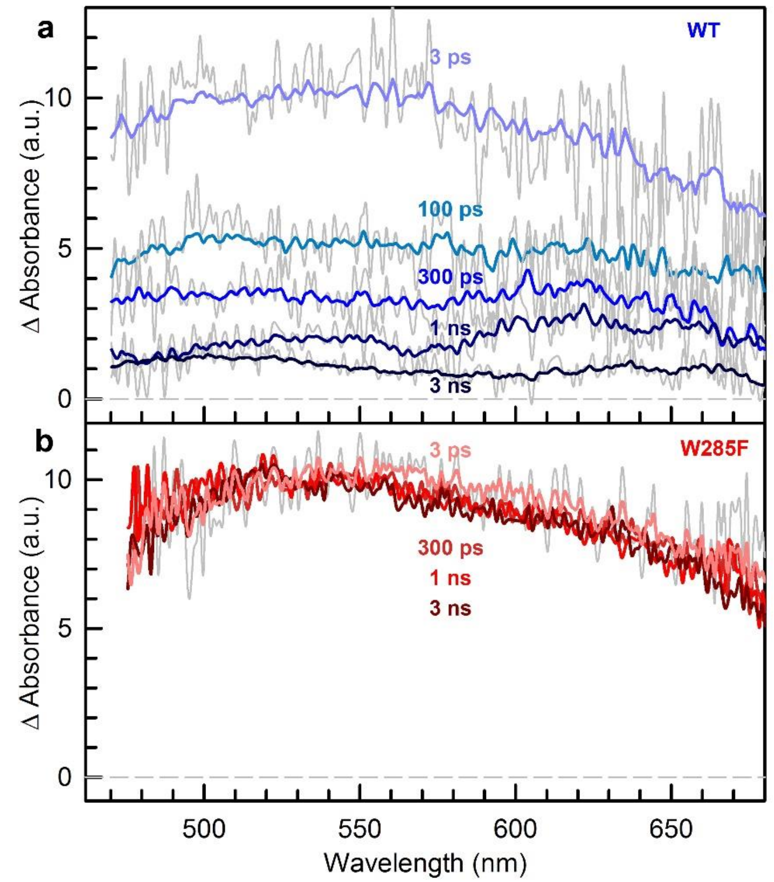


**Supplementary Fig. 5 | Femtosecond-resolved broadband absorption spectra (315-nm excitation) of WT dimer (a) and W285F (b).** Raw data were shown in grey lines and smoothed data were shown in color lines. Note that, for W285F, only 3-ps raw data were shown. The spectra of W285F at different time delays are very close. However, significant differences were observed in WT dimer, owing to formation of tryptophan cationic radicals (about 630 nm) and Trp neutral radicals (about 500 nm).

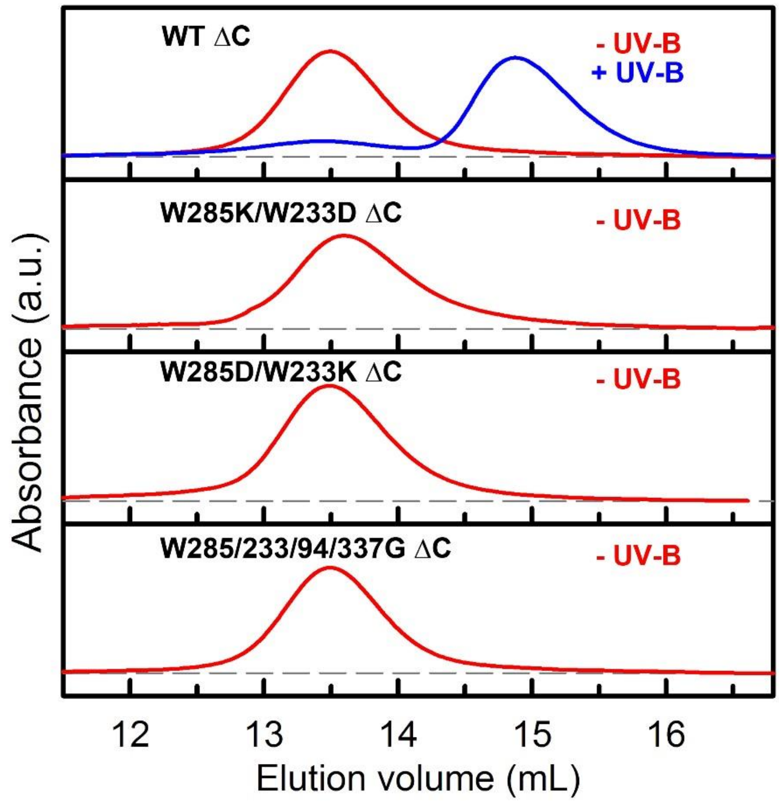


**Supplementary Fig. 6 | Size exclusion chromatography results for C-terminal truncated (ΔC) samples.** Note that W285K/W233D, W285D/W233K and W285/233/94/337G are constitutive dimers with no dissociation function.

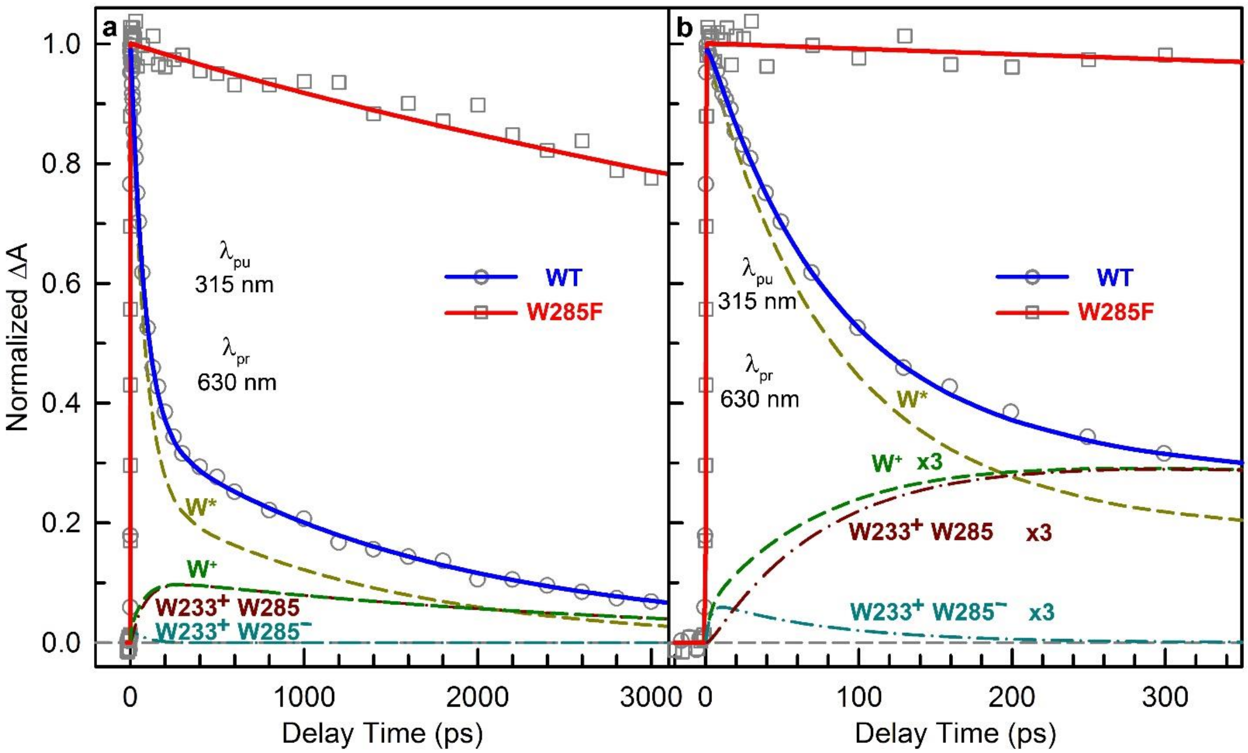


**Supplementary Fig. 7 | Single wavelength transient absorption data and contributions from different intermediates**. Pump wavelength ($\lambda_{pu}$) is 315 nm and probe wavelength ($\lambda_{pr}$) is 630 nm for WT (raw data: circles; fitting line: blue line) and W285F (raw data: quares; fitting line: red line). For WT, the transient was deconvoluted into Trp excited state ($W^*$ dark yellow dashed line) and Trp cation radicals ($W^{+\bullet}$ green dashed line) contributions. $W^{+\bullet}$ signal is further decomposed into $W233^{+\bullet}W285$ (dark red dash-dot line) and $W233^{+\bullet}W285^{-\bullet}$ (cyan dash-dot line) contributions. **a**, Full range data up to 3 ns. **b**, The first 350 ps data is shown. $W^{+\bullet}$, $W233^{+\bullet}W285$ and $W233^{+\bullet}W285^{-\bullet}$ signal was multiplied by 3. Note the initial ~3.2-ps rise in WT.

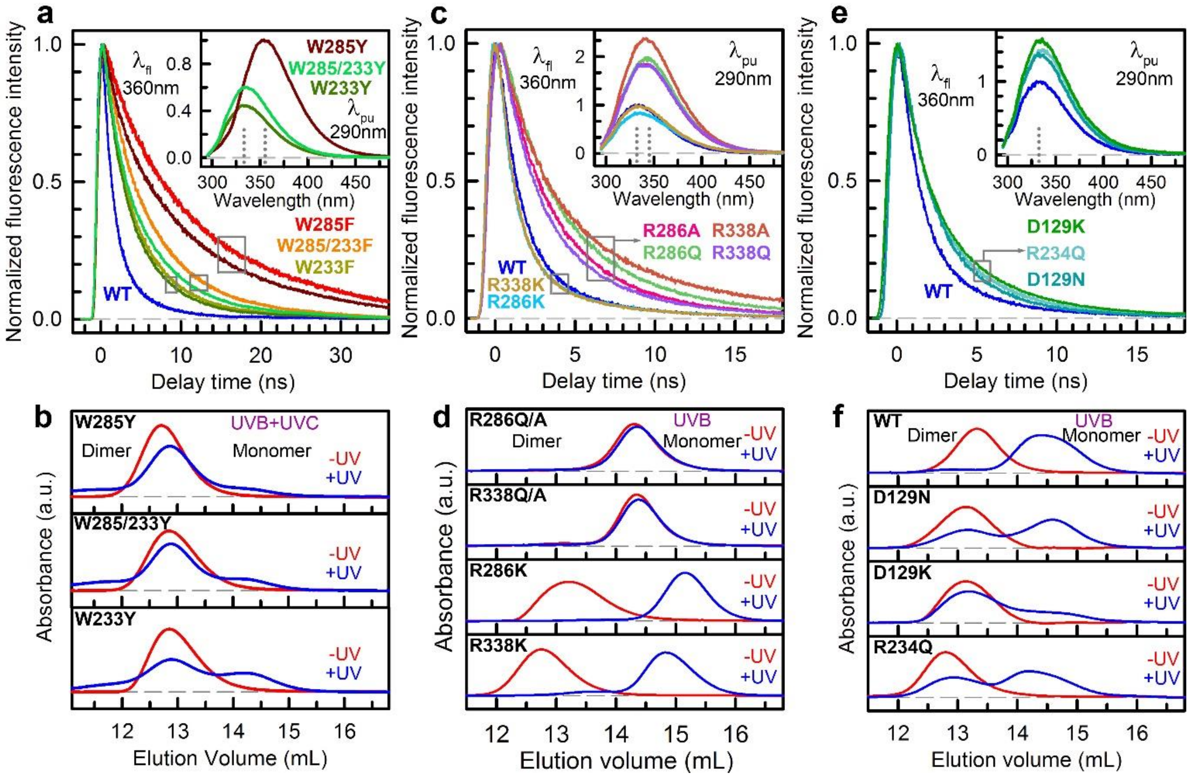


**Supplementary Fig. 8 | Mutant spectra, critical mutants, electron-transfer direction and dissociation function. a**, Fluorescence transients of various mutants of W285 and/or W233 to F/Y. The inset shows steady-state emission spectra of various mutants with emission peaks marked by dotted lines (334 nm and 354 nm). **b**, Size exclusion chromatography (SEC) profiles before (red) or after (blue) 30 minutes of UV-B+UV-C irradiation for W285Y, W285/233Y and W233Y. **c**, Fluorescence transients and steady-state emission spectra (inset) of critical R286 and R338 mutants marked with emission peaks by dotted lines (332 nm and 343 nm). **d**, SEC results before (red) or after (blue) 10 minutes of UV-B irradiation for R286 and R338 mutants. **e**, Fluorescence transients and steady-state emission spectra (inset) of WT, D129N, D129K and R234Q with the marked emission peaks by a dotted line (332 nm). **f**, SEC results before (red) or after (blue) 10 minutes of UV-B irradiation for WT, D129N, D129K and R234Q.

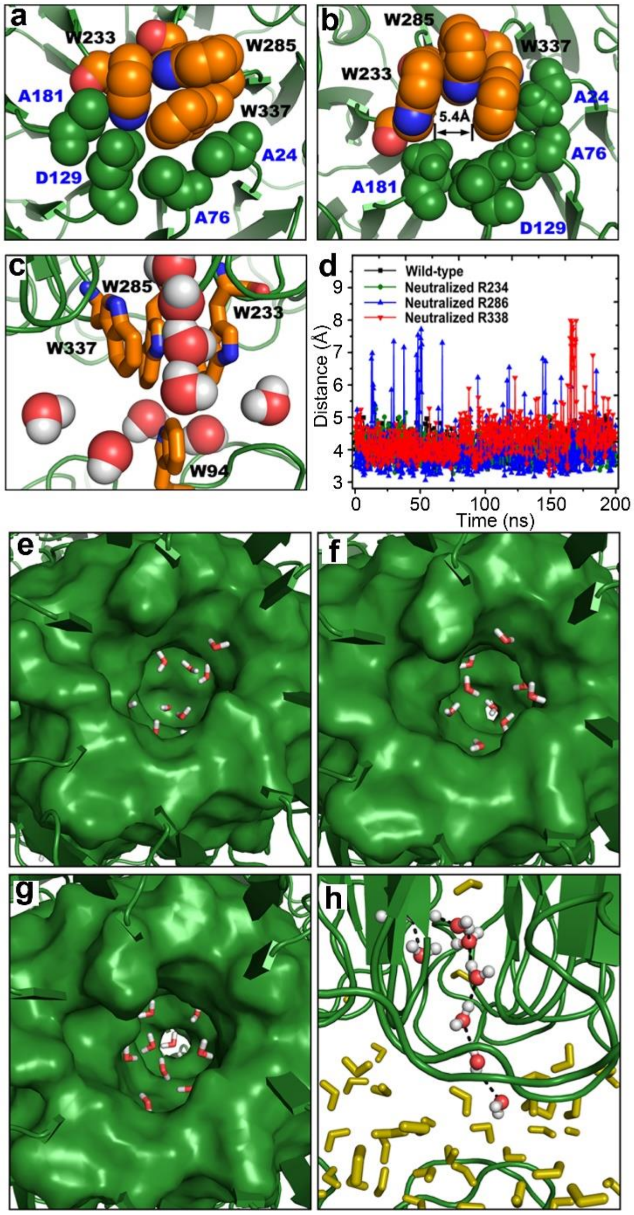


**Supplementary Fig. 9 | The gate connecting the dimer interface and the interior water channel is formed by W233, W285 and W337. a**, A top view of the water gate in the closed state. **b**, A top view of the water gate in the open state. A separation of 5.4 Å between W233 and W337 was observed in the open state. **c**, The channel water and interfacial water molecules are in direct contact and exchangeable when the gate is open. **d**, A histogram of the inter-tryptophan distance along the molecular-dynamics trajectory. The R286-neutralized and the R338-neutralized states were observed to have open state (large distances). For parent state and R234-neutralized state, the gate remains closed during the 2-μs simulation. **e**, A bottom view of the water channel in closed state. **f**, Water channel is partially open in an R286-neutralized snapshot. **g**, Water channel open in an R286-neutralized snapshot. **h**, Channel water molecules (in spheres) and interfacial water molecules (yellow sticks) can exchange via the opened gate.

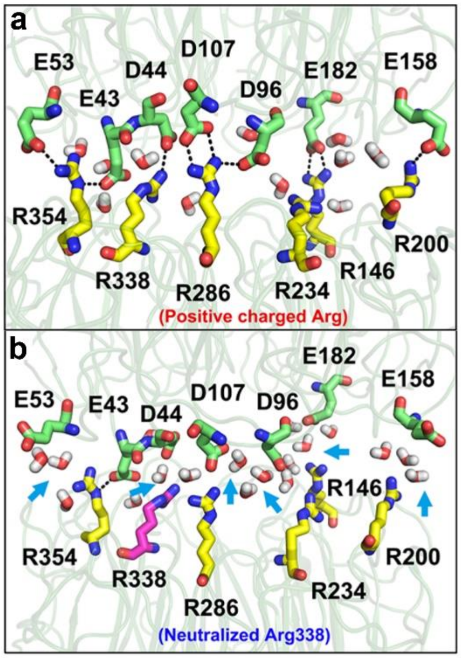


**Supplementary Fig. 10 | Snapshot configurations from molecular dynamics simulations illustrating interfacial salt-bridge interactions for the dark state of UVR8 dimer (a), and the activated state with R338 neutralization (b).** For clarity, only key salt bridges are shown from one side of the interface. Key salt bridges across the dimer interface found in the X-ray crystal structures are mostly maintained during molecular dynamics simulation of the dark state. Several interfacial salt bridges are interrupted, forming solvent-separated ion pairs or fully dissociated ions. Major changes in ion-pair interactions in the arginine-neutralized configurations are indicated by light-blue arrows.

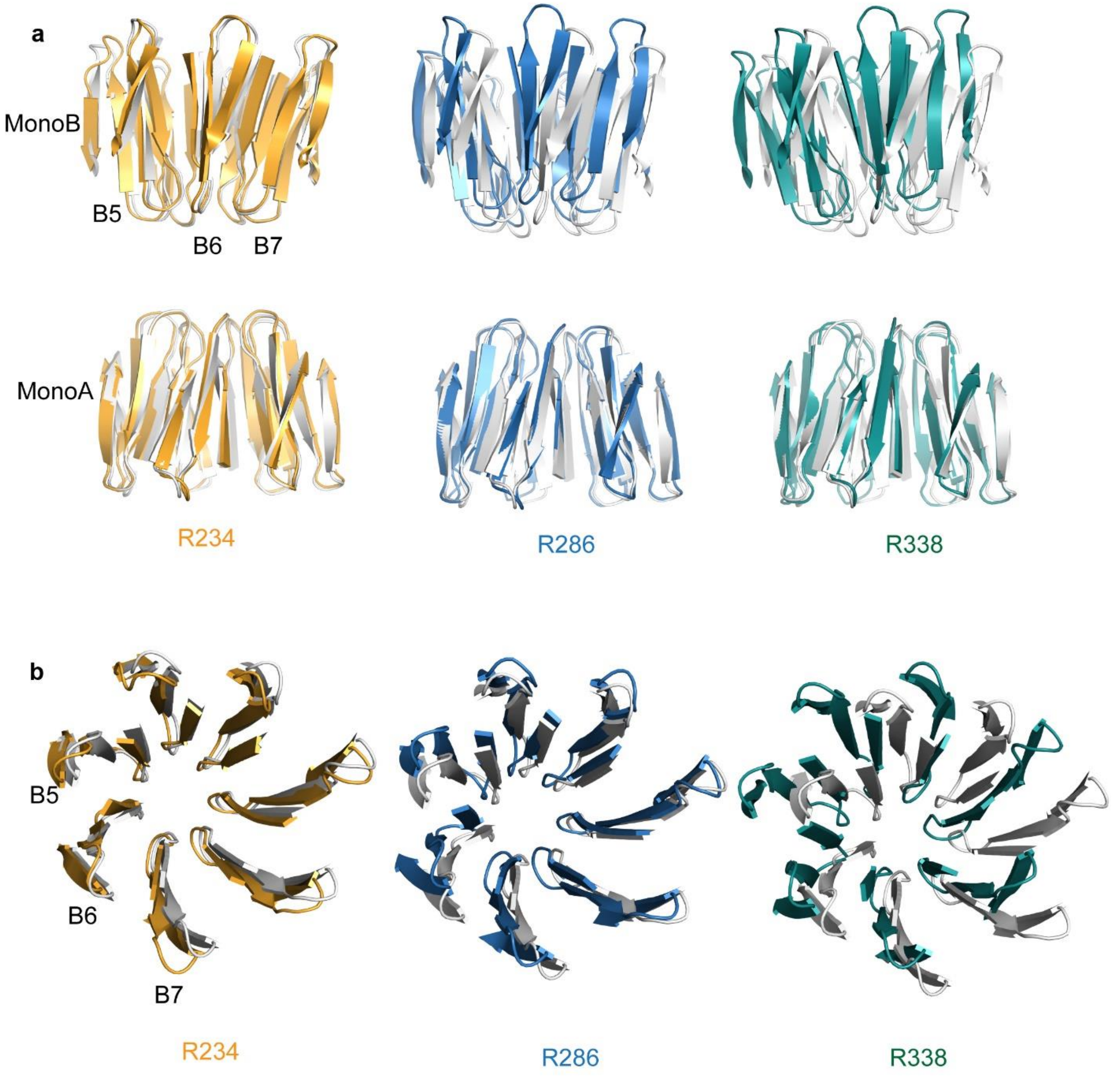


**Supplementary Fig. 11 | Typical MD snapshot structures from neutralized R234 (yellow), neutralized R286 (blue) and neutralized R338 (cyan), comparing with a parent state snapshot structure (grey). a**, Side view. **b**, Top view. The ground and neutralized R234 are very stable, while neutralized R286 and R338 make large conformational changes. The most prominent structural changes occur in blades 5 and 6, where W285 and W233 are located, respectively. An unwinding of the dimer was seen in neutralized R286 and R338 structures.

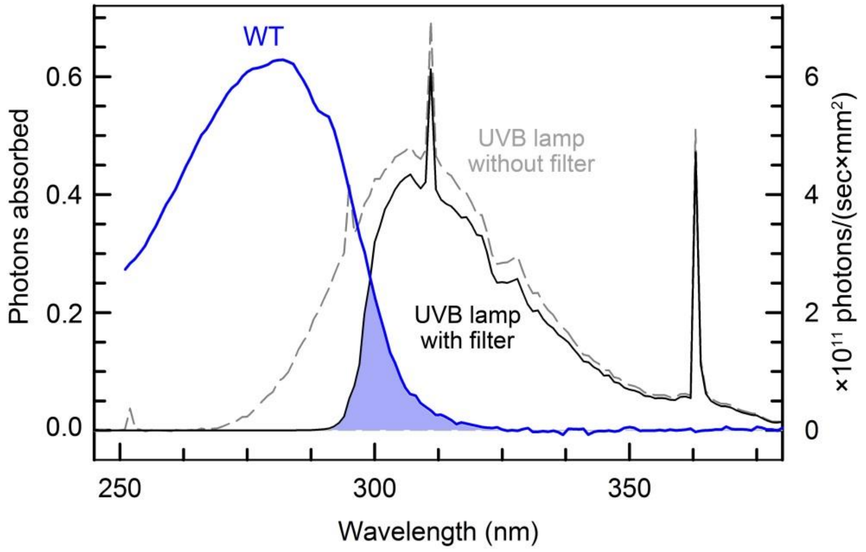


**Supplementary Fig. 12 | Spectrum of UV light (with the filter, black line; without the filter, grey dashed line) in units of photon flux (right axis) and ratio of photons absorbed (left axis) by UVR8 sample.** With the filter, we estimated that about 3.9 photons were absorbed for each UVR8 dimer molecule per minute.

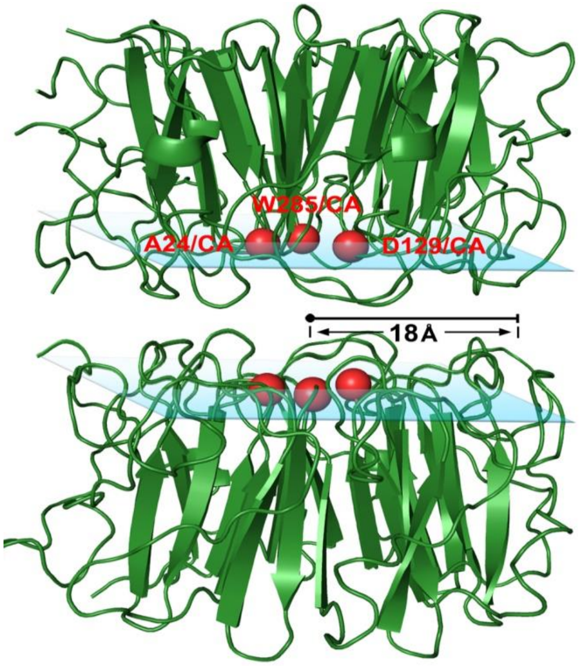


**Supplementary Fig. 13 | Definition of the interface region for water number counting.** Carbon-$\alpha$ atoms of W285, D129 and A24 are shown in red. The two planes defined by the 3 carbon-$\alpha$ atoms are shown in cyan. Number of water molecules in between the two planes, and within 18 Å of the center of mass of the 6 carbon-$\alpha$ atoms was counted.

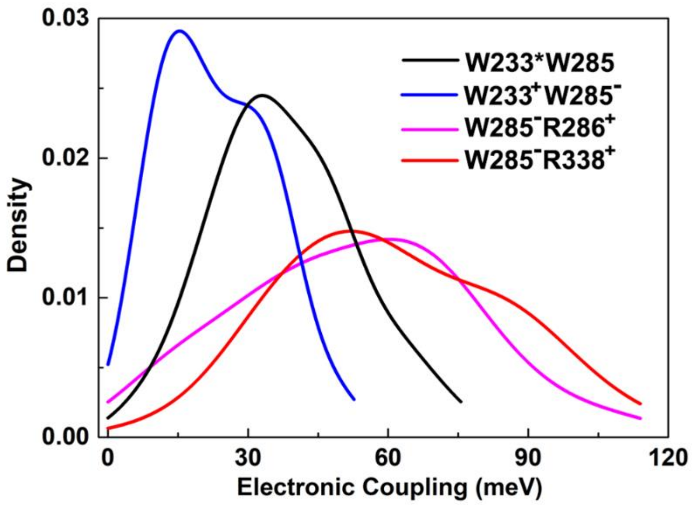


**Supplementary Fig. 14 | Distribution of the computed electronic coupling matrix elements for the electron transfer reactions from the excited W233* and to W285 (black), from W285$^{-\bullet}$ radical to W233$^{+\bullet}$ radical (blue) and from W285$^{-\bullet}$ radical to R286 (pink) and to R338 (red).** The transient intermediate W285 anion radical plays a role as an electron transfer hopping site from excited W233* to distant arginine residues. Due to instantaneous variations of donor and acceptor distance and dynamic fluctuations of the surrounding protein environment, the computed electronic coupling for each electron transfer process spans a range of 100 meV. The average electronic coupling values are listed in Supplementary Table 3, which are used to compute the rates of electron transfer.

## References

1. Li, X. *et al*. A leap in quantum efficiency through light harvesting in photoreceptor UVR8. *Nature Commun.* **11**, 1-9 (2020).

2. Peon, J., Hess, G. C., Pecourt, J. L., Yuzawa, T. & Kohler, B. Ultrafast photoionization dynamics of indole in water. *J. Phys. Chem. A* **103**, 2460-2466 (1999).

3. Yanai, T., Tew, D. P. & Handy, N. C. A new hybrid exchange–correlation functional using the Coulomb-attenuating method (CAM-B3LYP). *Chem. Phys. Lett.* **393**, 51-57 (2004).

4. Adamo, C. & Barone, V. Toward reliable density functional methods without adjustable parameters: the PBE0 model. *J. Chem. Phys.* **110**, 6158-6170 (1999).

5. Schmidt, M. W. *et al*. General atomic and molecular electronic structure system. *J. Comput. Chem.* **14**, 1347-1363 (1993).

6. Chan, W. -L. *et al*. The quantum coherent mechanism for singlet fission: Experiment and theory. *Acc. Chem. Res.* **46**, 1321-1329 (2013).

7. Hush, N. Homogeneous and heterogeneous optical and thermal electron transfer. *Electrochim. Acta*. **13**, 1005−1023 (1968).

8. Marcus, R. A. Electron transfer reactions in chemistry. Theory and experiment. *Rev. Mod. Phys.* **65**, 599−610 (1993).

9. Marcus, R. A. & Sutin, N. Electron transfer reactions in chemistry: theory and experiment (Nobel Lecture). *Angew. Chem., Int. Ed. Engl.* **32**, 1111−1121 (1993).

10. Siddarth, P. & Marcus, R. A. Electron-transfer reactions in proteins: an artificial intelligence approach to electronic coupling. *J. Phys. Chem.* **97**, 2400−2405 (1993).

11. Åqvist, J., Medina, C. & Samuelsson, J. -E. A new method for predicting binding affinity in computer-aided drug design. *Protein Eng. Des. Sel.* **7**, 385-391 (1994).

12. Ren, H. S., Provorse, M. R., Bao, P., Qu, Z. X. & Gao, J. L. Multistate density functional theory for effective diabatic electronic coupling. *J. Phys. Chem. Lett.* **7**, 2286-2293 (2016).

13. Gao, J. L., Grofe, A., Ren, H. S. & Bao, P. Beyond Kohn Sham approximation: hybrid multistate wave function and density functional theory. *J. Phys. Chem. Lett.* **7**, 5143-5149 (2016).
